# Porcine ear necrosis in nursery piglets is preceded by oral manipulations of the ear

**DOI:** 10.1186/s40813-024-00388-4

**Published:** 2024-11-13

**Authors:** Mateusz Malik, Koen Chiers, Ilias Chantziaras, Dominiek Maes

**Affiliations:** 1 Department of Internal Medicine, Reproduction and Population Medicine, Faculty of Veterinary Medicine, Ghent University, Ghent, Belgium; 2 Department of Pathobiology, Pharmacology and Zoological Medicine, Faculty of Veterinary Medicine, Ghent University, Ghent, Belgium

**Keywords:** Porcine ear necrosis, Nursery pigs, Behaviour, Ear biting, Tail biting, Oral manipulations, Chewing

## Abstract

**Background:**

Porcine ear necrosis (PEN) is characterized by dry crusts on the ear tip. The crusts often progress to moist and bloody lesions and may lead to partial loss of the ear tissue. The cause and pathophysiology of PEN are unknown. Skin infections, systemic infections, or ear biting have been suggested as a cause of PEN, but no proper evidence has been shown. The behavioural factor has not yet been investigated, therefore this study evaluated the importance of oral manipulations in the occurrence of PEN in nursery pigs. Three farms affected by PEN were visited weekly, and the prevalence and severity were recorded. Video recordings of the animals were performed, and the behaviour was evaluated. The presence of pathogens in the lesions and histological alterations were also analysed.

**Results:**

The highest percentage of pigs with PEN lesions in the farms ranged between 58 and 93%, with most lesions being of mild to moderate severity. The first ear lesions occurred about 1–2 weeks after an increase in the number of ear manipulations in the pens. The frequency of the ear manipulations clearly changed over time, and the number of oral ear manipulation behaviour significantly differed (*P* < 0.05) between pigs in pens with high and low PEN prevalence. Increased ear manipulation behaviour was significantly related to a subsequent increase in PEN lesions (OR = 4.3; *P* < 0.001). Metagenomic investigation of lesion scrapings revealed a variety of pathogens mostly with low abundance, where microscopic alterations were found mainly in the epidermis.

**Conclusions:**

Oral manipulation of the ear pinnae by pen mates was followed by the development of PEN lesions one to two weeks later. This suggests that the behaviour played an important role in the PEN lesions formation in the nursery pigs of the three farms. Bacteria found in PEN lesions most probably were secondary to initial external skin damage, but their relevance needs to be investigated further.

**Supplementary Information:**

The online version contains supplementary material available at 10.1186/s40813-024-00388-4.

## Introduction

Porcine ear necrosis (PEN) can be described as dry crusts on the tip of the ear, which may develop into wet, bloody lesions and cause a partial loss of ear tissue. This condition occurs worldwide and mainly affects pigs after weaning [1–4]. The condition named porcine necrotic ear syndrome was described for the first time in 1984 [1], and the authors suggested using this name only temporarily until the aetiology and pathogenesis were specified. However, the term ‘‘syndrome’’ might not be appropriate, as this refers to a group of clinical signs or lesions, whereas in the case of PEN, ear lesions are the only sign. Forty years later, PEN has neither been fully reproduced yet, nor has the pathogenesis been elucidated. Researchers reported infections with pathogens such as *Staphylococcus hyicus* and *Staphylococcus aureus* as potential causes of PEN [1, 5]. Others suggested ischemic necrosis due to cold agglutinin formation during *Mycoplasma suis* infection as a pathway of the lesions [6]. Also, a significant association between ear biting, high air humidity and ear necrosis was found [5]. However, none of these studies provided evidence regarding the trigger for early lesion formation. In our previous studies [7, 8], no correlation to ear lesions could be determined despite the analysis of several factors, including antibodies, antigens originating from bacteria or viruses, presence of mycotoxins in the feed and blood, air temperature and humidity, or pathomorphological investigation of the lesions. However, in the latter study, lesions were less severe on ears with an ear tag (paced close to the ear margin, not in the middle of the ear), suggesting that this location of ear tags reduces the ear area available to be bitten (mechanical protection) and the possible importance of ear biting in the pathogenesis. The histological investigation of affected ears in that study [8] also revealed alterations in the upper layers of the skin. Additionally, common factors causing tail and ear biting were suggested [9, 10], where tail biting is recognized as the main cause of tail wounds [11]. A very recent study [12] associated PEN with increased duration of oral manipulations of the pen mates, and decreased time of nosing. It is reported that weaning and mixing animals can be a major stress factor for piglets [13]. Agonistic behaviours aiming at different body parts were observed after the animal’s regrouping at weaning [14], and the enrichment of the environment may also influence the behaviour of pigs [15], as chewing objects is a typical way of exploring the environment, which in barren conditions can be redirected to pen mates [16, 17].

We hypothesized that ear oral manipulations (chewing/ pulling/ biting) are an important triggering factor for PEN lesions. Therefore, the main aim of the present study was to evaluate the importance of oral manipulations in the occurrence of PEN in nursery pigs. Secondly, for the comparison with our previous study, the presence of pathogens in ear scrapings and sera were analysed, histopathology of the ear biopsies was performed, and additional air quality parameters were measured. Finally, the tail manipulations and lesions were evaluated.

## Materials and methods

The study protocol was approved by the Ethical Committee of the Faculty of Veterinary Medicine and the Faculty of Bioscience Engineering, Ghent University (EC2022-034), as well as by the Flemish governmental agency for animal welfare (DWZ/LD/22/1.15/70).

### Farm characteristics and study design

Three commercial single-site farrow-to-finish pig farms that were struggling with PEN in nursery pigs, were included in the study. On farms A and C, recurrent problems with tail and flank lesions in the nursery pigs were reported. Farm A housed 400 Hypor (Large White x Landrace) sows, farm B housed 320 German Genetics sows, whereas, on farm C, 190 sows were present of DanBred and Hypor genetics. The health status of the animals was routinely inspected by the herd veterinarian, and in all three farms, piglets were vaccinated before the weaning against porcine reproductive and respiratory syndrome virus (PRRSV), porcine circovirus type 2 (PCV2), and *Mycoplasma hyopneumoniae*.

On farm A piglets were weaned at four weeks of age and two litters were merged in one pen (25 piglets), on farms B and C, mixing by weight after weaning was performed at four and three weeks of age, respectively. The tails were docked on all farms, on farm A they were shorter (3–4 cm) than on the other two farms (ca. 6 cm). The following enrichment materials were the only ones provided in each pen of the nursery units: Farm A- two wood pieces of ca. 7 × 7x25cm hung on chains, Farm B- two chains of ca. 30 cm covered by rubber, Farm C- two pieces of PVC pipe 10–15 cm, hung on chains. The ratio of enrichment material to pig was 1:12.5; 1:16.5; and 1:18.5 on farms A, B, and C respectively. On farm A, fully slatted plastic flooring was used, on the other farms, in between plastic slats, there was a solid part with floor heating covering 12–15% of the floor area. The floor area available for the animals was 0.38 m^2^, 0.33 m^2^, and 0.27 m^2^ per pig, on farms A, B, and C, respectively. In all farms, ad libitum dry feed was provided and drinking nipples were used. The number of feeding places and drinking nipples per pen was 10 and 4 on farm A, 6 and 6 on farm B, and 4 and 4 on farm C. The drinking water on farms A and C was decontaminated with hydrogen peroxide.

The study was conducted between 16th September and 8th November 2022 on farms A and C, and between 11th January and 22nd February 2023 on farm B. On each farm, one batch of piglets was followed from weaning to the end of the nursery, spanning 6–7 weeks. The first author visited the farms to collect appropriate data and samples. The numbers of pens monitored during the study were as follows in the three farms: Farm A- 20 pens (496 pigs) in one compartment, Farm B- 14 pens (473 pigs) in one compartment, Farm C- 12 pens (446 pigs) in three compartments, with the average number of pigs/pen 25, 32, and 37 for farms A, B, and C respectively. The first visit occurred the day after weaning, with subsequent visits taking place at the end of each week post-weaning. This resulted in eight visits to farms A and C, and seven visits to farm B. During the visits, the animals were inspected by entering the pen, after video recordings were taken.

### Prevalence and severity of PEN

**Fig. 1:**
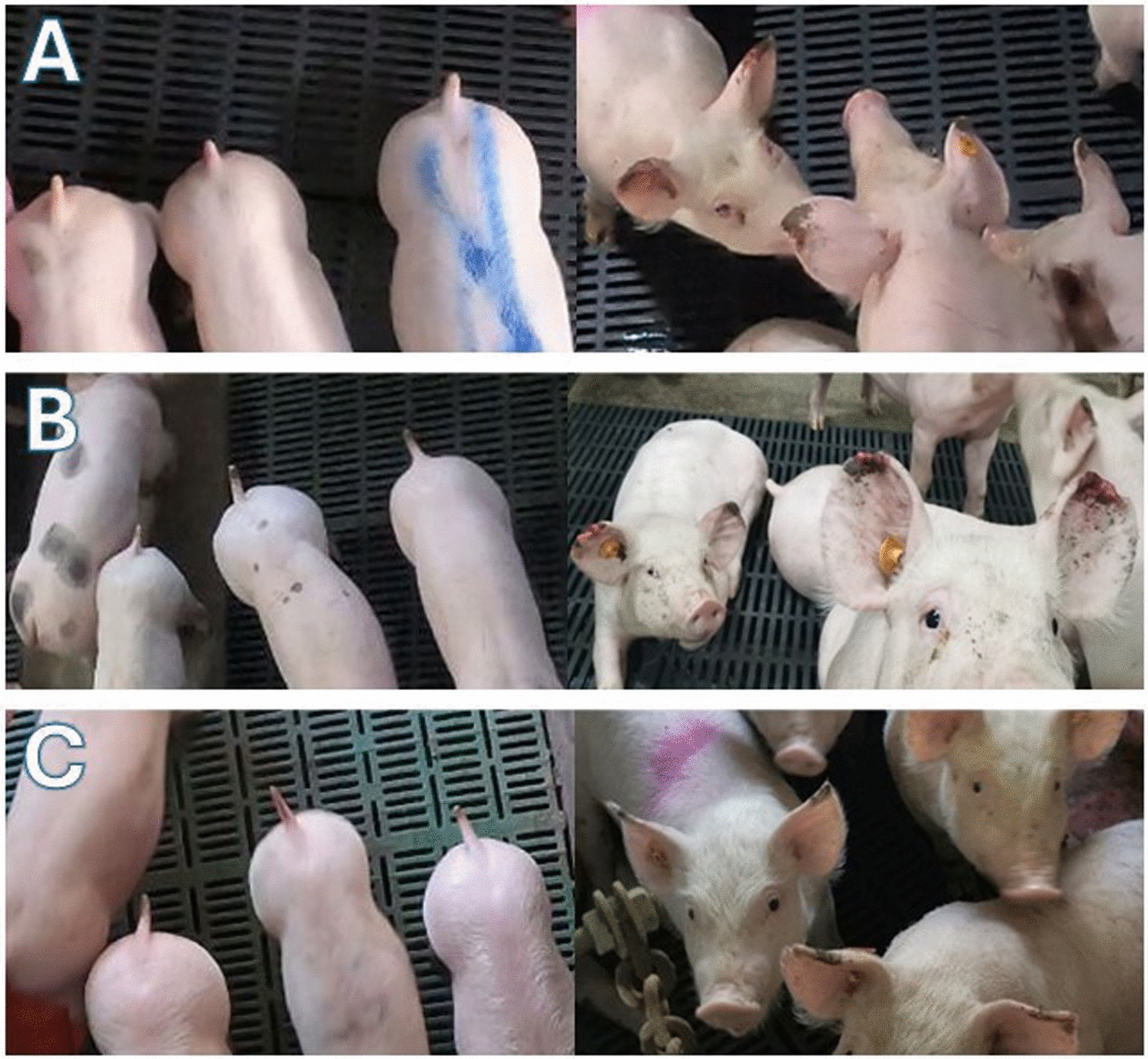
Examples of tail length, tail and ear lesions of the piglets on farms** A**,** B** and** C**. Ear lesions scored 1- Farm A and C, ear lesions score 2- farm B. Ear lesions with scores 1 and 2 were present in all three farms. Tail lesions prevalence on farm B and C was 19% and 22%, respectively, and 2.6% on farm A

During each farm visit, all animals were visually evaluated in their pens for the presence and severity of PEN lesions and recorded at the pen level. The severity of the ear lesions was assessed using a five-point scoring system as described previously [7] with the examples of lesions presented in Fig. 1. In short, the scores (0 to 4) corresponded with the following conditions: 0 = no deviations, 1 = incipient red discoloration or a crust at the tip of the ear, 2 = more black-like discoloration, wound, and/or a rounded ear tip, moist, fresh, or with crusts, 3 = moist, bloody severe lesion with a part less than one-third of the ear missing, and 4 = loss more than one-third of the ear. In case pigs were affected on both ears, the score of the ear that was most severely affected was recorded. For comparative reasons, the prevalence of tail lesions, namely the presence of crust/ wound (without severity scoring), was recorded.

### Measuring the behaviour by video recording

Two mobile cameras- GoPro® 8 Black (GoPro Inc., USA) were used for the recordings. They were placed about 2 m above floor level to capture the entire pen, and the piglets in each pen were video recorded during each herd visit for 17.5 min. The recording always began at 0800 AM, but to avoid capturing the same pen at the same time each day, the order of the video-recorded pens was changed each time. While recording, the operator was not present in the compartment, so that the animals were not distracted. The first 2.5 min of each video recording were excluded from behavioural analysis, allowing the animals to return to normal behaviour after the placement of the cameras by the operator. The length of the excluded section of the video is based on the maximum duration for a single video file (17.5 min). This was found adequate based on prior evaluations of multiple videos. The following 15 min of the video were used to evaluate the events of oral ear manipulations- including biting, chewing, or pulling. Additionally, for comparative reasons, the oral manipulations of the tail (biting, chewing, pulling) were assessed.

### Sampling

To perform the analyses on animals with different severity of lesions, samples were collected in the second last week of the nursery period. On each farm, 15 affected (different lesion severity in different pens) and 5 non-affected animals were chosen, and the blood samples were taken via jugular vein puncture. Individual samples of five animals with the same severity of lesions were pooled into one sample, ending-up with 4 pooled samples from each farm.

From the 20 animals that were blood sampled within each farm, 10 were selected to obtain scrapings of the ears: 6 from affected (different lesion severity) and 4 from non-affected animals. The scrapings of the lesions and underlying tissue from PEN-affected animals, and ear skin scrapings from unaffected animals, were collected with a bistoury knife.

Additionally, on the same sampling day, to allow comparison with the previous study [8], ear biopsies were taken for histopathological examination. To this end, on each farm, five animals with ear scrapings and with different ear lesion scores, were selected. From the affected animals (n = 3), the biopsy was taken from the margin of the lesion (from the non-scraped ear area). From non-affected animals (n = 2), the biopsy was taken from the ear tip, 5–10 mm from the ear edge. A six-millimetre disposable dermal biopsy punch tool (Kai Medical) was used.

### Metagenomic analysis

The pooled serum samples and the scrapings were analysed using nanopore metagenomic sequencing in the PathoSense® laboratory, at the veterinary faculty of Gent University. The serum samples were pre-purified using a patented sampler (EP 19183233.6). The scrapings were crushed in dPBS using a 1.5 mL Eppendorf tube squisher (Zymo H1001) and filtered through a 1.5 mL Eppendorf tube 0,8 µm centrifuge filter (Vivaclear, Sartorius) at 2,000 rpm for 5 min.

The resulting filtrates were subjected to enzymatic host depletion and an *ad random* amplification procedure. The resulting DNA was subjected to rapid library preparation using the RBK096 library prep (ONT), multiplexing up to 24 samples per run. Sequencing was performed on R9.4.1 flow cells (ONT) using the GridION device, which allows real-time data acquisition, super accurate base calling, and demultiplexing via Guppy (v.6.1.5). The reads were taxonomically classified using in-house validated bioinformatics pipelines. A spike-in virus was used to perform normalization between samples and to give a semi-quantitative report as described before [18]. This allowed us to report both, viruses and bacteria, in five abundance categories, namely: very low, low, medium, high, and very high. The classification of bacteria was limited to the genus level as previously discussed [19]. If two or fewer absolute reads were identified, they were not included in the report [18].

### Histopathology

The collected samples for histopathology were first fixed in 4% neutral buffered formalin and embedded in paraffin before being stained with haematoxylin and eosin followed by microscopic evaluation by a pathologist certified by the European Board of Veterinary Specialisation. Investigated samples included lesions scored 0–2.

### Air quality parameters

On each farm, one Healthy Climate Monitor® device was placed in the middle of the compartment, at a height of 2 m above the floor. The device measured the following air quality parameters every 30 min throughout the entire nursery period: ambient temperature (°C), relative humidity (%), and concentration of CO_2_ and NH_3_ (ppm).

### Data analysis

Pens for the behavioural evaluation were chosen considering the average prevalence of PEN during the nursery phase (6–7 weeks). On each farm, five pens with the highest mean prevalence of PEN (40–80%) (high PEN prevalence- HPP) were selected. The selection of pens with a low prevalence of ear lesions (LPP) was determined based on a mean PEN prevalence below 20%. On farm A, there were five LPP pens, for farms B and C, there were only one and two pens with a low prevalence, respectively. The behaviour analysis was performed only in these selected pens, therefore the total duration of all evaluated videos was 44.5 h; 20 h for farm A, 10.5 h for farm B, and 14 h for farm C.

All statistical analyses were performed using IBM SPSS version 29® (Armonk, New York, USA). Descriptive information regarding the various parameters included in this study was calculated for all farms and for each farm separately. For data analysis, the number of affected piglets was selected as the dependent variable. Upon inspection, the data were found to be over-dispersed. Hence, we used a negative binomial with log-link count model with pen included as subject and week as repeated factor. Farm and “ear manipulations events” (binary variable) were included as fixed factors. Pairwise comparisons were run post-hoc for the two groups (HPP and LPP) and statistically evaluated with Wald chi-square statistic. Differences were declared as statistically significant if *P* ≤ 0.05.

## Results

### Prevalence of PEN lesions

**Fig. 2:**
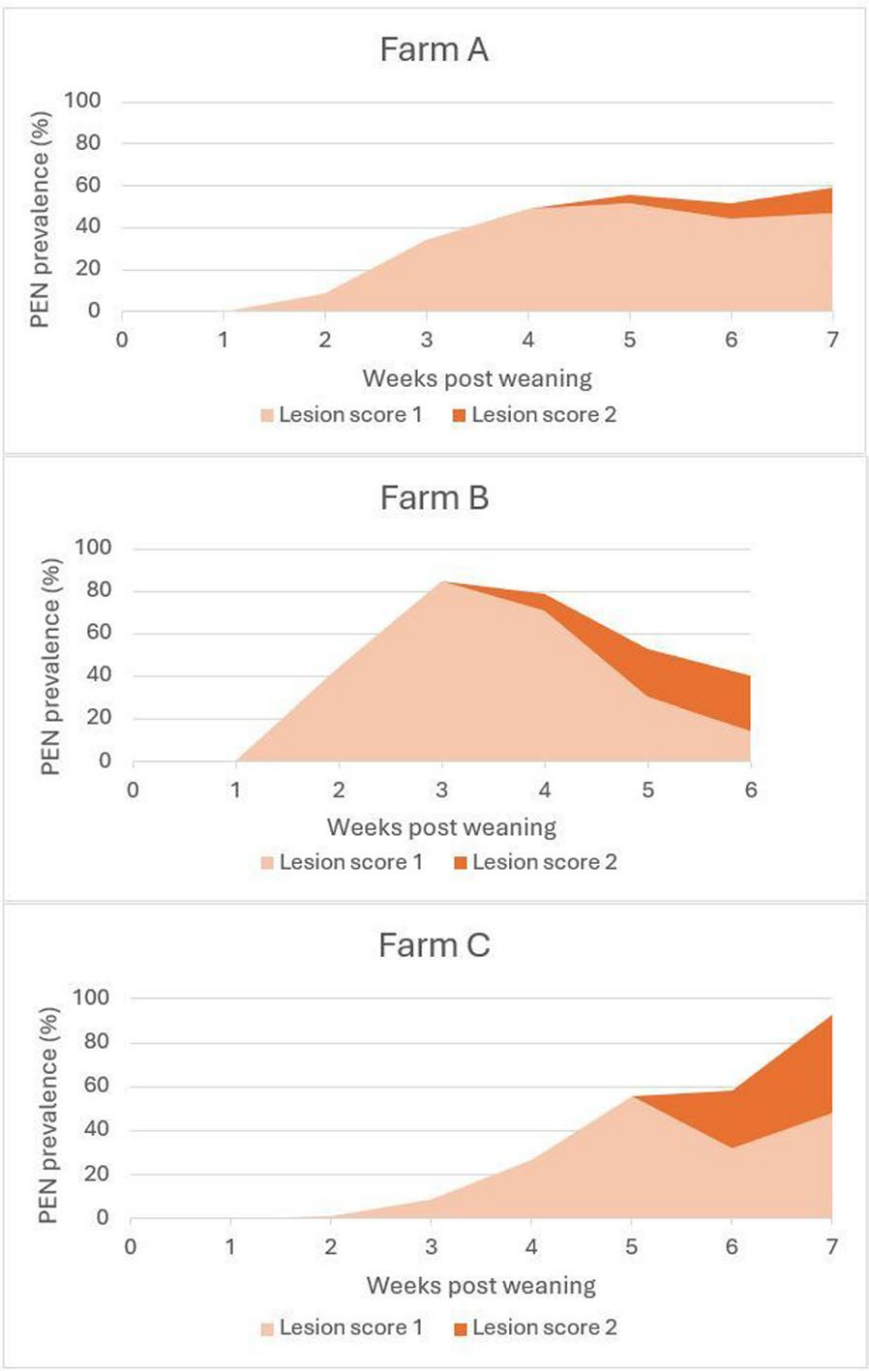
The prevalence of porcine ear necrosis (PEN) lesion scores 1 (incipient red discoloration or a crust at the tip of the ear) or 2 (more black-like discoloration, wound, and/or a rounded ear tip, moist, fresh, or with crusts) was recorded once a week during the nursery period (6–7 weeks) on the three farms A, B, and C. Number of piglets included: Farm A: n = 496, Farm B: n = 473, Farm C: n = 446

The weaning groups included in the study comprised 496, 473, and 446 piglets on farms A, B, and C, respectively, and no animals showed signs of PEN on the first day after weaning. The prevalence of pigs affected by PEN in farms A and C increased with the number of weeks post-weaning and reached a maximum of 58% and 93%, respectively, in the last week of the nursery period. The maximum prevalence on farm B was 93% and occurred in week 3 post-weaning, and thereafter, the prevalence decreased to 40% at the end of the nursery period. Figure 2 depicts the total weekly prevalence of pigs with PEN lesions in farms A, B, and C. There were no pens without affected animals.

### Severity of the PEN lesions

The severity of the lesions was assessed during the visits. The first lesions appeared as mild, but with time, the first lesions with a score of 2 started to appear in the second half of the nursery period. At the end of the nursery period, the percentage of pigs with a score of 1 was 46%, 14%, and 48% in farms A, B, and C, respectively. The percentage of pigs with a score of 2 was 12%, 26%, and 45% in farms A, B, and C, respectively. Pigs with a score of 3 or 4 were not found.

### Location of HPP and LPP pens in the compartment

The maximum prevalence of affected animals per pen ranged from 12 to 100%. On farm A, more HPP pens were located in the front of the compartment where the windows and doors were placed. On the other hand, more LPP pens were in the back of the compartment (no windows). On farm B, the only LPP pen was in the front of the unit (entrance and windows) whereas PEN prevalence was similar in the other pens. On farm C, where study animals were housed in three small compartments, the two LPP pens were close to the doors, but away from the windows.

### Ear manipulation behaviour

The total duration of recordings captured on farm A was 47 h, on farm B 29 h, and on farm C 28 h. The different durations were related to the different number of pens on each farm. On all farms, in the HPP pens, pigs performed an average between 133.4 ± 18.3 to 165.2 ± 17.6 ear manipulations per pen during all observations. Conversely, in the LPP pens, the numbers ranged between 59 ± 0 and 109.2 ± 36.6. This corresponded to an average number of ear manipulations/ pig/ 15 min in HPP pens 0.51–0.87 versus 0.31–0.51 in LPP pens.

The full results related to oral ear manipulations in HPP and LPP are presented in Table 1.

**Table 1:** The number of ear manipulation events based on video recordings of nursery pigs in pens with high- (HPP) and low- (LPP) porcine ear necrosis (PEN) prevalence in farms A, B, and C

		Farm		
		A	B	C
Duration of nursery period (weeks)		7	6	7
No. of observations (15 min)		8	7	8
Average no. of pigs/pen		25	33	37
No. of pens (total no. of piglets)	HPP	5 (120)	5 (168)	5 (186)
	LPP	5 (134)	1 (27)	2 (73)
Total no. of oral manipulations recorded in the pens in all observations	HPP	826	667	760
	LPP	546	59	204
The average no. ± SD of oral manipulations/ pen in all observations	HPP	165.2 ± 17.6	133.4 ± 18.3	152.0 ± 21.4
	LPP	109.2 ± 36.6	59 ± 0	102.0 ± 3.0
The average no. ± SD of oral manipulations/ pen / 15 min	HPP	20.6 ± 9.2	19.0 ± 7.5	19.0 ± 6.1
	LPP	13.6 ± 4.1	8.4 ± 5.7	12.7 ± 6.2
The average no. of oral manipulations/ pig / 15 min	HPP	0.87	0.57	0.51
	LPP	0.51	0.31	0.35

### Associations between ear manipulation behaviour, and the prevalence of PEN lesions

In all three farms, oral manipulations of the ears were observed already on day 1 post-weaning, both in HPP and LPP pens. The highest number of manipulations on farms A and B took place in weeks 2 and 1 post-weaning respectively, reaching 171 manipulations/ pen/ observation. On farm C, the maximum value (152) occurred in week 6 post-weaning, with a lower peak (123) in week 2. On every farm, in LPP pens, the number of oral manipulations per pig was lower.

**Fig. 3:**
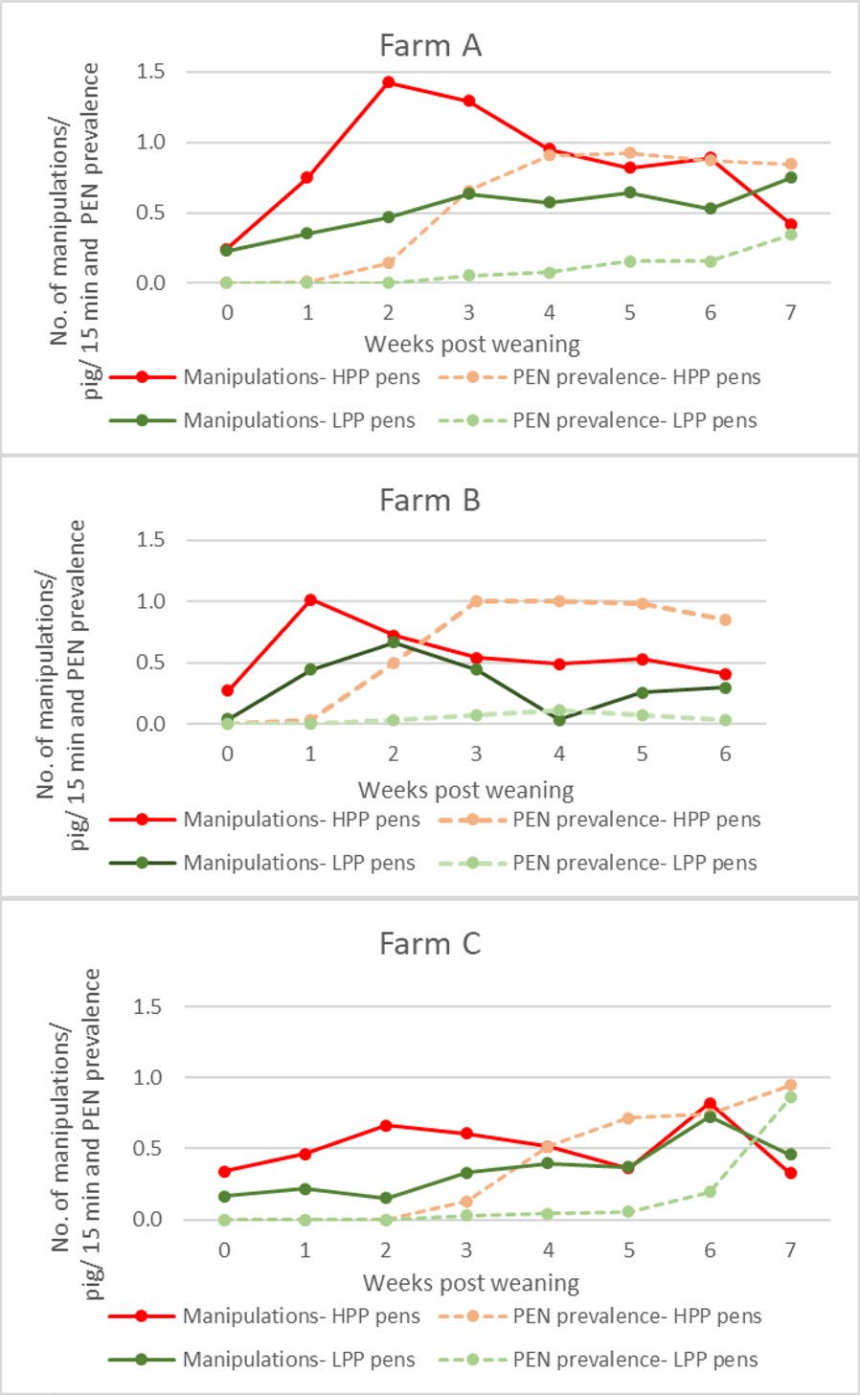
The number of ear manipulation events/ pig/ 15 min and porcine ear necrosis (PEN) lesion prevalence in pens with high PEN prevalence (HPP), and pens with low PEN prevalence (LPP), during the entire nursery period on farms A-C. Number of piglets included: Farm A: HPP n = 120, LPP n = 134; Farm B: HPP n = 168, LPP n = 27; Farm C: HPP n = 186, LPP n = 73

The frequency of ear manipulations changed over time (Fig. 3). On all three farms, the average number of ear manipulations/pig/15 min was higher in HPP pens compared to LPP pens. The odds of developing an ear lesion in HPP pen was 4.29 (95% Cl 2.80 to 6.56) times higher than for pigs in the LPP pens (*p* < 0.001). Additionally, the increase in manipulation frequency was higher and also faster in HPP pens than in LPP pens.

The lesions occurred about one week after the increase in oral manipulations.

### Tail versus ear manipulations and tail lesions

In all three farms, the number of tail manipulations/pig/15 min recorded for each observation in HPP and LPP pens was below 0.12, except for week 2 on farm B when the value reached 0.222. The average number of manipulations/pig/15 min of all observations in HPP versus LPP pens were: 0.078 versus 0.067 (Farm A), 0.051 versus 0.074 (Farm B) 0.066 versus 0.053 (Farm C).

**Fig. 4:**
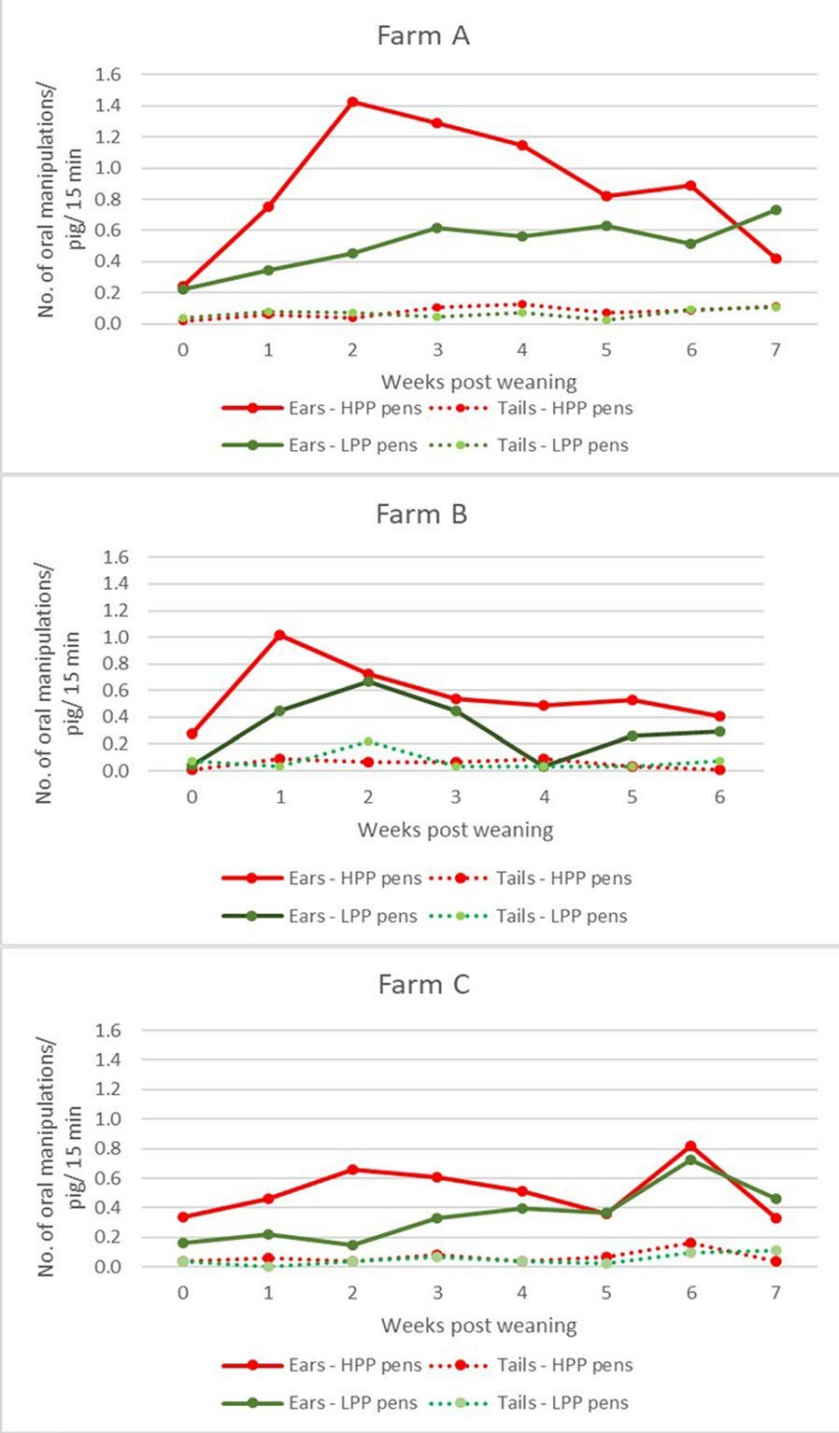
Graphical presentation of the number of ear and tail manipulations/pig/ in pens with high PEN prevalence (HPP), and pens with low PEN prevalence (LPP), during the entire nursery period on farms A-C. Number of piglets included in the evaluation: Farm A: n = 120, Farm B: n = 168, Farm C: n = 186

The full data on the number of tail and ear manipulations/pig/15 min are shown in Fig. 4.

On Farm A, the highest percentage of pigs affected by tail lesions in HPP pens was 2.5%. The value was reached after a gradual increase from week 3 post-weaning. In the LPP pens the maximum prevalence of 3% was reached in the fourth week post weaning, and deceased to 2% in the last two weeks. On farm B, the first tail alterations in HPP pens were recognized in the second week after weaning, and from then onwards, gradually more animals were affected, reaching 19% pigs in the last week, where in the LPP pens first tail lesions appeared in the third week post weaning, reaching 18% at the end of the study. On farm C, in HPP pens 2% of the animals developed tail lesions in the first week post-weaning. This percentage reached its maximum of 22% prevalence at the end of the nursery period, whereas in LPP pens first lesions were recognised in the second week after weaning, affecting 18% of piglets at the end of the nursery period. Photos of the affected tails are presented in Fig. 1.

### Metagenomic analyses

Overall, several viruses and bacteria were detected in the serum using nanopore metagenomic sequencing, mostly at very low levels. Torque teno sus virus (TTSuV) and PRRSV were found in all three farms. The frequently found bacteria (positive samples/all samples) in the serum of the three farms, were *Pseudomonas* sp*.* (9/12), *Staphylococcus* sp. (6/12), and *Corynebacterium* sp*.* (5/12). The bacterial loads were very low.

In the scrapings, astrovirus, bocaparvovirus, picobirnavirus, rotavirus C, porcine torovirus, and rotavirus B were the most prevalent viruses. They could be extracted in samples from every farm in PEN-affected and non-affected pigs. Bacteria found in the highest number of scrapings were *Streptococcus* sp*., Staphylococcus* sp*., Clostridium* sp*., Moraxella* sp.*, Limosilactobacillus* sp.*, Rothia* sp*., and Fusobacterium* sp*.* They were present in all three farms, but farm C had the least number of positive samples. Two bacteria, namely *Fusobacterium* sp*.* and *Mycoplasma hyopharyngis* (*M. hyopharyngis*) were present mainly in scrapings from lesions but not in the scrapings from the healthy skin. The full table of pathogens analysed by metagenomic analysis is presented in the Additional file 1.

### Histopathology

**Fig. 5:**
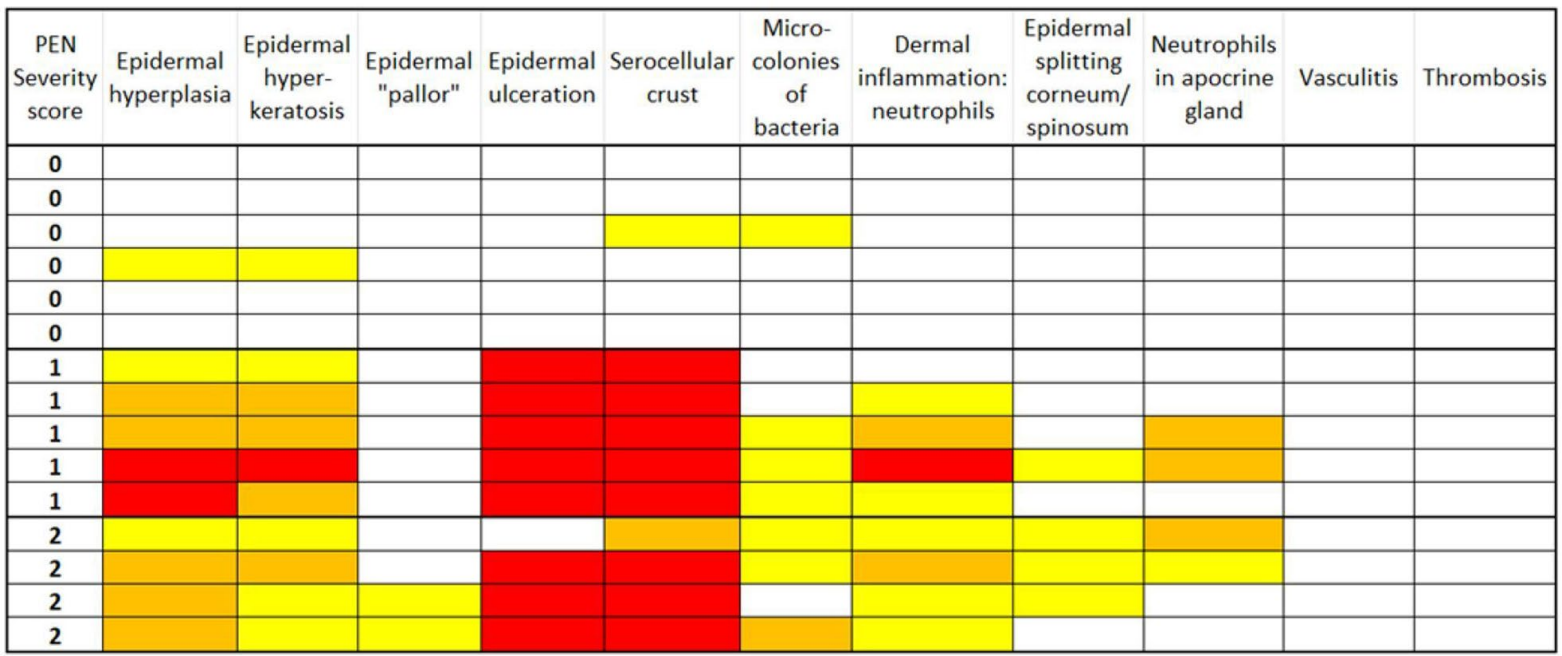
Heatmap of histopathological findings in ear biopsies of piglets with different severity scores of ear lesions (score 0 = no deviations, 1 = incipient red discoloration or a crust at the tip of the ear, 2 = more black-like discoloration, wound, and a rounded ear tip, moist, fresh, or with crusts). Cells marked in yellow correspond to mild alterations, cells marked in orange- moderate alterations, and red cells- severe alterations for each of the histopathological parameters

The most prevalent tissue alterations were detected in the epidermis, namely hyperplasia (10/15), hyperkeratosis (10/15), and ulceration (8/15) together with the presence of serocellular crust (10/15). These lesions were found almost exclusively in samples of ears with lesions. Bacterial coccoid microcolonies (7/15) and neutrophils (8/15) were observed only in samples of affected tissue. Vasculitis, thrombosis, dermal granulation of the tissue, and signs of skin necrosis were not present in any sample. The histopathological findings of all samples are summarized in Fig. 5.

### Air quality parameters

The temperature was the least fluctuating parameter (25–29 °C). The humidity fluctuated between 50 and 80% depending on the farm. The CO_2_ levels were above 3000 ppm for about 2.5 weeks on farm B, and on farm C for a short time in weeks 2 and 4. The recorded NH_3_ concentration also fluctuated between 5 and 40 ppm.

Precise climate data collected in the stables during the nursery period are presented in Additional file 2.

## Discussion

This study investigated the prevalence of PEN, and its association with oral ear manipulation behaviour in nursery pigs. Oral manipulations of the ear increased in the first week after weaning and were followed by the occurrence of ear lesions one to two weeks later. The odds of developing ear lesions in an HPP pen were 4.29 times higher than for pigs in the LPP pens (p < 0.001). Most lesions were mild, lesions of moderate severity mainly developed during the second half of the nursery period.

### Prevalence of PEN

The overall percentage of pigs affected by PEN in the present farms (58–93%) was comparable to prevalence reported by Kureljušić et al. 2021 [3] and Weissenbacher-Lang et al. 2012 [20], but they were higher than those found in our previous studies [7, 8]. Two different patterns of ear lesion development could be identified. The first one appeared aa slow but steady increase of the lesions and was observed in farms A and C, and the second one was characterized by a rapid increase post-weaning followed by a major drop two weeks later, and was observed in farm B. Besides our previous studies [7, 8] only one study recorded PEN lesions over time [21]. In that study, the development of lesions had a similar pattern as on farm B.

### Severity of lesions

Only mild (score 1) to moderate (score 2) lesions were observed. Most of the lesions were mild (score 1). Score 2 lesions gradually increased and were most prevalent in the second half of the nursery. This was also observed by other researchers [21]. Mild lesions were decreasing gradually during the second half of the nursery. This suggests that score 1 lesions may either evolve to score 2 lesions, or heal and disappear.

### Location of HPP and LPP pens in the compartment

The variation of lesion prevalence between different pens within a farm was relatively high. No housing characteristics could be identified, explaining the high prevalence of PEN lesions in some pens (HPP) versus the low prevalence in other pens (LPP). Within each farm, all the pens had the same size, type of floor, and pen partitions. This suggests that the housing conditions were likely not responsible for the difference between HPP and LPP pens.

On farm B, only the number of animals per pen was different between LPP and the other pens, namely 27 instead of 32–35. This finding agrees with another study [22], where pigs with more space allowance had fewer ear lesions. However, our finding should be interpreted carefully as it was the only LPP pen on farm B. However, it would be beneficial to investigate the space allowance factor in future studies. Based on our data, we could not draw definitive conclusions regarding the impact of pen location on the occurrence of ear lesions. On farm A, a higher number of HPP pens were situated closer to the windows (with more light) than at the back of the compartment, and this observation is interesting for future research.

### Ear manipulations

There was a clear change in the number of oral manipulations of the ears over time. In all three farms, the frequency of the behaviour increased during the first half of the nursery period. Similar observations were already shown in other publications [23, 24]. In one study [23], the manipulative behaviours peaked in week three post-weaning. In the other study [24], pigs were evaluated for three weeks, and a comparison was made in manipulative behaviour between piglets that were weaned and moved to a nursery unit, versus piglets that stayed with the sows in pens with straw. The piglets in the nursery unit performed significantly more massaging and suckling on other piglets and objects than pigs remaining with the sow. Whether weaning piglets at a young age followed by redirected suckling behaviour, played a role in our study is unknown, but the results do not point in that direction. Piglets on farm C were weaned at a younger age (3 weeks) than piglets on farms A and B, but they performed fewer ear manipulations. Further research is warranted to assess the effect of weaning age in piglets on manipulative behaviour post-weaning.

The slatted floors in the nurseries could have contributed to the occurrence of oral behaviours redirected toward pen mates [25]. Slatted flooring is considered a barren environment. It does not encourage interaction with the surroundings or does not satisfy the pigs' need for rooting and chewing [26]. Enrichment materials were present in the three farms, but they might have been less effective as they lacked complexity, destructibility, or edibility [27], and due to the low amount of these materials (ratio material:pig of 1:12.5–18.5) animals could have had a limited possibility to interact with them. Besides wood, the chains and plastic tubes also do not fully align with the characteristics of good enrichment materials according to EU legislation [28]. The same enrichment materials were present across pens within each farm, therefore the results do not demonstrate the role of environment enrichment in the development of ear lesions. Further research is necessary to explore this factor.

It has been suggested that ear and tail biting may “spread” to other pigs via visual communication [29]. Therefore, it can be speculated that redirected exploratory behaviour (oral manipulations) towards pen mates [24] combined with such visual communication could explain the high prevalence of oral manipulations observed in our study. The average number of ear manipulations per pig per 15 min ranged between 0.31 and 0.51 in LPP pens, and between 0.51 and 0.87 in HPP pens. These values are in line with values (recalculated to 15 min) reported by other researchers, namely 0.25–0,5 [30] or 0.9–2.4 [31]. These authors also reported the presence of ear lesions in the animals.

### Relation between ear manipulations and ear lesions

There was a significant difference in the number of oral earl manipulations of animals in HPP and LPP pens. The positive value of the odds ratio (4.29) (*p* < 0.001) indicates that pigs housed in HPP pens had a significantly higher risk of developing PEN lesions one week later compared to pigs housed in LPP pens. This illustrates the role of oral manipulations in the development of PEN lesions. Repeated manipulations could mechanically damage the epidermis of the skin, creating a potential entry point for bacteria additionally altering the epidermis. In all farms, oral manipulations occurred immediately after weaning and before the first ear lesions appeared. Interestingly, a rapid increase in the manipulations during the first week (Farms A and B) coincided with the appearance of the first lesions during the second week. The association was somewhat different in farm C, where there was a slower increase in the number of manipulations post-weaning, and lesions developed in the third week post-weaning. In week 6 of farm C, the number of manipulations in HPP and LPP pens (for unknown reasons) increased, leading to a higher prevalence of lesions one week later. Therefore, the present study shows that ear manipulations may serve as a trigger for PEN lesion development. This has been suggested but not proven many decades ago [1]. However, further studies are needed to evaluate the potential significance of bacterial secondary skin infections in PEN pathogenesis.

### Tail versus ear manipulations and tail lesions

The number of tail manipulations remained constant and was lower than the number of ear manipulations. No major changes in the frequency of tail manipulations were noticed during the nursery period compared to ear manipulations. However, manipulations focus on one tail, whereas ear manipulations are directed to two ears. Speculatively, this lower amount of tail manipulations could explain the lower tail lesion prevalence. The significantly lower prevalence of tail lesions in farm A, compared to farms B and C, could be explained by the fact that the tails were docked shorter in that farm, making it more difficult for the pigs to reach the tail by the teeth. It was shown that finisher pigs with short docked tails (2.9 cm) had a lower risk of being bitten on the tail than pigs with 5.7 cm, 7.5 cm or undocked tails [32]. The lower prevalence of tail lesions in nursery pigs is also in line with other studies in grower-finisher pigs with docked tails [33, 34]. The latter authors suggested that ear lesions mainly occur in younger animals (e.g. nursery pigs), whereas tail lesions are more prevalent in older animals (e.g. fattening pigs) [33, 34].

Schrøder-Petersen and colleagues showed an increase in the frequency of oral manipulations of tails shortly after weaning [35]. They speculated that, under certain conditions, the frequency and intensity of oral manipulations can exceed a threshold, resulting in lesions. Applying this to our data on ear manipulations, a theoretical threshold value for ear manipulations per pig per 15 min can be established, above which pigs develop ear lesions one week later. For the three farms, these values range between 0.5 and 0.6. Based on these similarities between the results [35] and our findings, the primary formation of ear lesions likely has a similar pathogenesis. However, this did not apply to our data on tail manipulations, which were consistent over time, and tail lesions developed gradually and much slower than the ear lesions.

### Metagenomic analyses

Overall, different viruses and bacteria were detected in the sera using nanopore metagenomic sequencing, but mostly at very low levels. Similar results were obtained in a previous study [8].

Several viruses were detected in the ear scrapings. As they were found in ears with and without PEN lesions, these viruses likely play only minor role, if any, in the development of PEN lesions. This was also suggested by de Costa et al. 2021 [4].

Staphylococci and streptococci were present in high numbers in ear scrapings. However, their primary role is also questionable, since they were found in scrapings of both affected and non-affected animals. Interestingly, *M. hyopharyngis*, and *Fusobacterium sp*. were present only in scrapings of affected ears. *Mycoplasma hyopharyngis* has been isolated from the oropharynx of pigs, but their pathogenic potential has not yet been demonstrated [36]. This bacterium was already demonstrated in ear lesions [8] which could indicate its involvement in the pathogenesis. However, they might be secondary contaminants because of the oral manipulations of the ear. *Fusobacterium necrophorum* is an anaerobic bacterium. Itis commonly found in the environment and the oral cavity of pigs and is known for its potential to invade damaged skin and mucosa [37].

### Gross lesions and histopathology

The gross and the histopathological lesions were similar between the three farms, as well as to those obtained in a previous study [8]. The lack of any vasculopathy, in both mild and moderate lesions suggests that the lesions did not develop from the inside towards the outside. This strengthens our hypothesis about the importance of ear manipulations for the development of PEN lesions.

In addition, the histopathological features are similar to those observed in frictional dermatoses in humans [38]. This type of dermatitis is referred to as irritant contact dermatitis, a type of inflammation of the skin that occurs when it encounters a physical agent that directly damages the skin [39]. Extensive frictions damage the skin, creating an entry point for secondary bacterial infections [40], which was possibly also the case in our study. Finally, it was reported that moisture may increase the negative effect of friction lesions [39], where the moistening of the skin with saliva also occurs during oral manipulations (chewing) of the ears. This emphasizes the similarities between frictional dermatosis in humans and PEN lesions and the involvement of ear manipulations by pen mates in the pathogenesis of PEN. However, further research is required to confirm this theory.

### Air quality parameters

Although the temperature and humidity ranged within the recommended values for nursery pigs [41], the CO_2_ and NH_3_ levels fluctuated highly over time. Therefore, further research is needed to investigate any potential effects of air parameters on the formation of PEN lesions.

## Conclusions

The study showed a strong and significant association between oral manipulations of the ear, and the occurrence of PEN lesions one to two weeks later, suggesting that oral manipulations on the ear pinnae predispose to the development of PEN. The oral manipulations likely moisten and damage the skin, where the secondary bacterial invaders may play additional role in the further development of PEN. More research on the reasons for the oral manipulations, also in farms without PEN problems, is warranted to develop control and prevention measures and ultimately improve the health and welfare of nursery pigs.

## Supplementary Information

Additional file 1. Full results of the metagenomic analysis performed on ear scrapings of affected and non-affected pigs.

Additional file 2. Graphical presentation of the climate parameters (temperature-red, relative humidity-blue, CO2-yellow and NH3 levels-pink) during the entire nursery period for farms A-C.

## Data Availability

The datasets gathered and analysed during the current study are available from the corresponding author on reasonable request.
