# Supplementary figures and images for: Porcine ear necrosis in nursery piglets is preceded by oral manipulations of the ear
Source: Porcine Health Manag. 2024 Nov 13;10:51. doi: 10.1186/s40813-024-00388-4 (PMC11562591; doi:10.1186/s40813-024-00388-4)

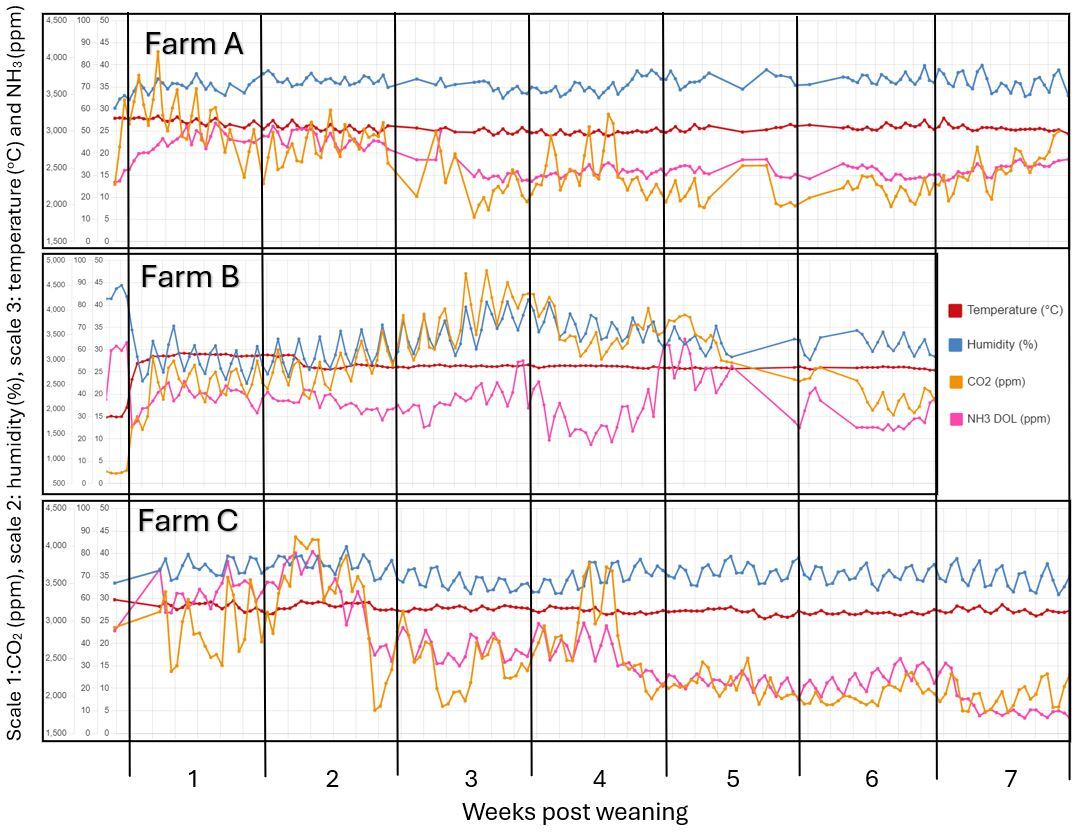

Supplement: Supplementary file 2 — Additional file 2. Graphical presentation of the climate parameters (temperature-red, relative humidity-blue, CO2-yellow and NH3 levels-pink) during the entire nursery period for farms A-C. [file 40813_2024_388_MOESM2_ESM.jpg]
